# P-6. Outcomes of Early Oral Antibiotic Treatment for Uncomplicated Enterococcal Bacteremia

**DOI:** 10.1093/ofid/ofaf695.237

**Published:** 2026-01-11

**Authors:** Madeline Schultze, Tiffany Wu, Alexander Rock, Eric M Gillett, Cecilia Li, Alyssa R Letourneau

**Affiliations:** Massachusetts General Hospital, Boston, Massachusetts; Massachusetts General Hospital, Boston, Massachusetts; Massachusetts General Hospital, Boston, Massachusetts; Brigham and Women's Hospital, Cambridge , Massachusetts; Massachusetts General Hospital, Boston, Massachusetts; Massachusetts General Hospital, Boston, Massachusetts

## Abstract

**Background:**

*Enterococcus* spp. is a common cause of healthcare-associated bloodstream infections. Limited data exists on the efficacy and safety of oral antibiotic therapy for enterococcal bacteremia. This study compares the clinical outcomes of adults with uncomplicated enterococcal bacteremia treated with intravenous (IV) therapy versus an early transition to oral (PO) therapy.

**Methods:**

Retrospective cohort study of adult patients with *Enterococcus* spp. bacteremia between January 1, 2021 and August 1, 2024 at two tertiary medical centers. Patients hospitalized with uncomplicated enterococcal bacteremia with appropriate source control were included. Notable exclusion criteria include polymicrobial bacteremia, positive blood cultures for ≥ 48 hours, and evidence of invasive infection. The primary outcome was clinical cure at 90 days. Secondary outcomes included infection recurrence, hospital length of stay (LOS), treatment duration, and 90-day mortality. Safety outcomes, dosing strategies, and treatment durations were also evaluated.

**Results:**

73 patients were included. 35 patients were transitioned to PO therapy with linezolid (21/35, 60%) and amoxicillin (11/35, 31%) most commonly. Infection sources were predominantly intra-abdominal (41%) and genitourinary (35%). 26 (36%) patients were considered immunocompromised. Clinical cure rates were similar between the IV to PO vs. the IV group (88.6% vs. 81.6%; p=0.4). Infection recurrence was low between the two groups (5.3% vs. 7.9%; p = 1.0). The median antibiotic duration was comparable between the IV to PO vs. the IV group (14 days vs. 15 days; p=0.4). The median time to transition to PO therapy was 6 days [IQR, 3.5-8.5]. Hospital length of stay was significantly shorter in the IV to PO group compared to the IV group (9 days vs. 17 days; p=0.019). No significant difference was observed in 90-day mortality. Antibiotic-associated adverse effects were similar between groups.

**Conclusion:**

Clinical cure rates were comparable between the IV to PO and IV only group, and transitioning to PO antibiotics was associated with a shorter hospital LOS. These findings suggest that early transition to PO antibiotics may be a viable strategy for managing uncomplicated enterococcal bacteremia.

**Disclosures:**

All Authors: No reported disclosures

